# Prevalence of Stroke and Vascular Risk Factors in China: a Nationwide Community-based Study

**DOI:** 10.1038/s41598-017-06691-1

**Published:** 2017-07-25

**Authors:** Qi Li, Hao Wu, Wei Yue, Qingqing Dai, Hui Liang, Hetao Bian, Xiaoshuang Xia, Qiuhong Ji, Ying Shen

**Affiliations:** 1 Department of Neurology, the First Affiliated Hospital, Chongqing Medical University, Chongqing, China; 2 Department of Neurology, Tianjin Huanhu Hospital, Tianjin, China; 3 Department of Neurology, Affiliated Hospital of Guiyang Medical University, Guiyang, China; 4 Department of Neurology, the First Affiliated Hospital, College of Medicine, Zhejiang University, Hangzhou, China; 5 Department of Neurology, Jining No.1 People’s Hospital, Jining, China; 6 Department of Neurology, the Second Hospital of Tianjin Medical University, Tianjin, China; 7 Department of Neurology, Affiliated Hospital of Nantong University, Nantong, China; 8 Department of Traditional Chinese Medicine, Xuanwu Hospital of Capital Medical University, Beijing, China

## Abstract

We aimed to investigate the prevalence of stroke and related vascular risk factors in adult population aged 40 years and older in China. We conducted a prospective cross-sectional survey in nationally representative sample of 207323 individuals from all 31 Chinese provinces in 2013. Data were used to analyze the prevalence of stroke by age, sex, geographical regions and educational level. The age-standardized prevalence of stroke was significantly higher in men than in women in all age groups (P < 0.001). The age-standardized prevalence of stroke was significantly higher in rural than in urban residents among both men and women (P < 0.001). The prevalence of stroke was inversely associated with educational level. There were striking geographical variations in stroke prevalence in China with a higher prevalence of stroke in northern provinces as compared with southern provinces of the country. The age-standardized prevalence of hypertension, diabetes, dyslipidemia, atrial fibrillation and obesity in the Chinese population aged 40 years and older were 35.24%, 9.55%, 58.72%, 1.57% and 4.09%, respectively. Stroke and related vascular risk factors remains a major public threat in China and effective primary preventive strategies that aimed at reducing the burden of stroke and its risk factors are urgently needed.

## Introduction

Stroke is the second leading cause of death and a major cause of disability worldwide^1^. Globally, the burden of stroke increases significantly during the past two decades. Although the incidence of stroke has decreased in high-income countries, the burden of stroke increases significantly in low-income and middle-income countries^2^. China has a population of over 1.35 billion which accounts for one fifth of the world’s total population. The rapid economic growth and urbanization during the past 20 years has contributed to the dramatic change of the health profile in China^3^.

In 2010, stroke has replaced ischemic heart disease as the leading cause of death in China^3^. Although the stroke mortality rate has decreased, the incidence of stroke has increased significantly in China^4, 5^. Few studies have investigated the prevalence of stroke in China. A door-to-door stroke survey of 63192 residents from six cities showed that the age-adjusted prevalence of stroke was 719 per 100 000 in 1983^6^. A large scale population survey of 5 800,000 residents showed that the age-adjusted prevalence of stroke was 259.86 per 100,000 people in 1986^7^. However, these regional epidemiological studies were conducted in 1980s and cannot reflect the changing prevalence of stroke in China. Hypertension remains to be the most important risk factor for all types of stroke^8^. In addition, the prevalence of stroke risk factors remains unknown in the general population in China. The rapid economic growth and urbanization during the past 30 years has changed the lifestyle of Chinese residents and may lead to a changing burden of stroke in recent years. Investigation of the burden of stroke with its risk factors provides a more comprehensive assessment of the disease and is crucial for the development of effective policies for prevention and management of stroke in China.

In our study, we aim to investigate the prevalence and the risk factors of stroke in the adult Chinese population aged 40 years and older.

## Methods

### Study Population

The 2013 China Stroke Prevention Project (CSPP) was a national project, funded by the China Ministry of Finance, to investigate the prevalence and risk factors of stroke in a nationally representative sample of adult population aged 40 years or older. A total of 239553 residents aged ≥40 years within the 76 residential communities were invited to participate from 31 provinces in the study. A total of 208651 persons completed the survey and 1328 persons were excluded from the final analysis because of missing data. The study protocol was approved by the ethics committee of participating institutions and informed consent was obtained from participants. All methods were performed in accordance with the relevant guidelines and regulations.

### Data Collection

The organization and implementation of the CSPP was described before^9^. The research protocol for screening of stroke and risk factors include community based survey of prevalence of stroke, hypertension, diabetes mellitus, dyslipidemia, overweight and obesity, family history of stroke, smoking history, alcohol drinking history and atrial fibrillation in all participants. The CSPP program was implemented in 2 stages. The first stage of the CSPP program was conducted in local community centers. Residents aged ≥40 years were asked questions by trained medical staff using a standard questionnaire. The standard questionnaire included the demographics, past medical history, family history of stroke, drug use history, lifestyle and vascular risk factors. The educational background, marital status, occupation were also recorded. Body weight, height, waist circumference and blood pressure were measured and documented according to standard procedure. Participants with a history of stroke were carefully examined by trained neurologists on site to obtain more detailed information. A12-lead resting electrocardiogram was performed on site according to standard clinical protocol. The fasting plasma glucose and serum lipid panel including low-density lipoprotein cholesterol (LDL-C), high-density lipoprotein cholesterol (HDL-C), triglycerides and total cholesterol were tested and recorded. All participating staff are carefully-chosen by the project central office and are qualified to carry out high quality field study and community-based survey in China. All physicians who participated in the screening program are qualified professional and received adequate training to perform physical examination and data collection according to a standard protocol.

The project was organized and supervised by local governments to ensure high quality of the research. A central office for stroke prevention and management was established in Beijing and was responsible for training of examiners and providing technical assistance to strengthen their abilities to implement the survey. The study organizers have implemented a stringent quality assurance program to make sure the integrity and validity of the dataset. All investigators and physicians have received a standardized training program on the aim and protocol of the study before data collection. Staff and interviewers were trained on how to administer the survey using standardized questionnaire. Clinical physicians were trained to perform physical examination of participants according to a standard examination manual. Data were collected from each certified examination centers by trained medical staff and were reported through paper-based and web-based systems to the China stroke databank center in Beijing. The China stroke databank center staff checked the completeness and integrity of the data according to a standardized procedure.

### Definition of Stroke and Risk Factors

Stroke was defined according to the WHO criteria used in the WHO MONICA study as rapidly developing clinical signs of focal (or global) disturbance of cerebral function, lasting more than 24 hours or leading to death, with no apparent cause other than that of vascular origin^10^. Transient ischemic attacks were not included. The diagnosis of stroke was determined by clinical presentation and confirmation by computed tomography (CT) or magnetic resonance imaging(MRI) based on medical records. Hypertension was defined as systolic blood pressure ≥140 mm Hg, or diastolic blood pressure ≥90 mm Hg, and/or self-reported current treatment of hypertension with antihypertensive drugs^11^. Diabetes was defined based on WHO criteria with fasting plasma glucose ≥126 mg/dl (7.0 mmol/l), or self-reported previously diagnosed diabetes by healthcare professionals and/or receiving drug or insulin treatment for diabetes^12^. Smoking was defined as having smoked at least 1 cigarette per day in the last 3 months^13^. Alcohol intake was defined as drinking ≥100 mL spirit alcohol more than three times a week. Overweight was defined as a BMI ≥25 and <30 and obesity as a BMI of 30 or higher according to current criteria^14^. Based on the ATP III recommendations, dyslipidemia was defined as total cholesterol level of ≥5.18 mmol/L or low-density lipoprotein cholesterol ≥3.37 mmol/L or triglycerides ≥1.7 mmol/L in participants not taking lipid-lowering medications or if participants were treated with lipid-lowering drugs^15, 16^.

### Statistical Analysis

The 2010 sixth national population census of China was used as the standardized population to obtain national estimates. The stroke prevalence in the Chinese general population of people aged 40 years and older was estimated by age, gender, educational level, geographic region, and level of urbanization. All calculations were weighted to represent the population of Chinese people aged 40 years and older. The standardized prevalence of stroke was calculated for the overall population and for subgroups according to age, sex, level of urbanization, and educational level geographic region. A p value < 0.05 was considered statistically significant. All statistical analyses were conducted using SPSS version 19.0 software package.

## Results

### Prevalence of Stroke

**Figure 1:**
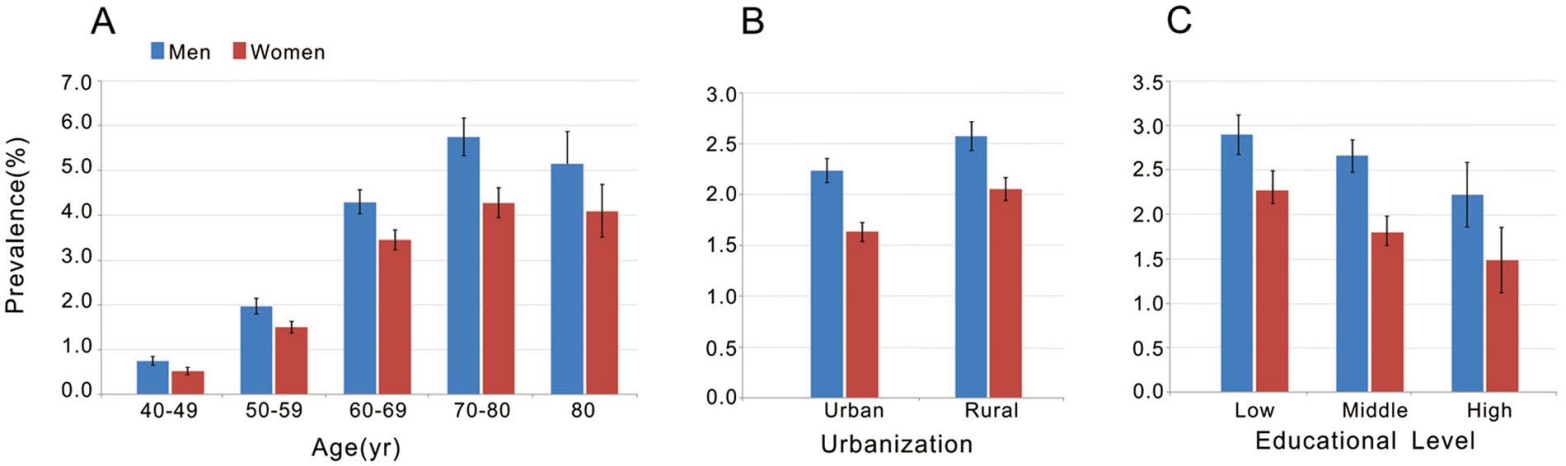
Age-Specific and Age-Standardized Prevalence of Stroke in Chinese Adults aged 40 Years and Older. The prevalence of stroke among men and women is illustrated according to age (Panel A), region of residence (Panel B) and educational level (Panel C). Educational level was classified according to national classification schemes as follows: low educational level indicates primary school education and below; middle educational level indicates secondary school education; high educational level indicates college education and above. Error bars (I) indicate 95% confidence intervals.

After excluding 1328 participants with missing values, 207323 individuals were included in the final analysis. Of these, 94913 (45.7%) were men and 112410 (54.2%) were women. A total of 114999 (55.4%) participants lived in urban regions and the remaining 92324 (44.5%) resided in rural areas. The mean age of participants was 57.72 years (age range 40–110 years). The general characteristics of and mean values of vascular risk factors of the study population are presented in Tables 1 and 2. The age-standardized prevalence was estimated to be 2.08% (95% CI, 2.02%-2.13%), 2.38% in men, and 1.82 in women. The age-standardized prevalence of stroke was significantly higher in men than in women in all age groups (P < 0.001). (Figure 1) Among individuals <80 years of age, the age-specific prevalence of stroke increased significantly with increasing age in both men and women (P < 0.001). The age-standardized prevalence of stroke was significantly higher in rural than in urban residents among both men and women (P < 0.001) (Fig. 1). The prevalence of stroke in people with low, middle and high educational levels were 2.50%, 2.22% and 1.94%, respectively (Fig. 1). Of the 5114 patients with stroke, 2380 (46.54%) were population of working age (<65 years old).

**Table 1:** General Characteristics of the Participants*.

Characteristics	Men	Women
Participants	94913	112410
Mean age (95% CI) — yr	57.50(57.42–57.57)	57.9(57.83–57.97)
Family history of stroke (95% CI) — %	5.70(5.55–5.85)	6.82(6.67–6.97)
College education or higher (95% CI) — %	13.97(13.81–14.14)	8.54(8.43–8.66)
Cigarette smoking (95% CI) — %	30.98(30.63–31.33)	2.20(2.11–2.28)
Consumption of alcohol (95% CI) — %	24.29(23.81–24.77)	2.68(2.52–2.85)
Urban residence (95% CI) — %	55.77(55.52–56.01)	55.22(55.00–55.44)
Mean body-mass index (95% CI)	24.17(24.13–24.22)	23.98(23.95–24.01)
Mean waist circumference (95% CI) — cm	84.55(84.49–84.61)	80.65(80.59–80.71)
Mean systolic blood pressure (95% CI) — mm Hg	130.17(130.07–130.28)	129.78(129.67–129.88)
Mean HDL cholesterol (95% CI) — mg/dl	55.47(55.32–55.63)	58.07(57.93–58.21)
Mean LDL cholesterol (95% CI) — mg/dl	110.60(110.40–110.80)	112.7(112.51–112.88)
Mean triglycerides (95% CI) — mg/dl	151.75(151.18–152.33)	144.59(144.14–145.04)
Mean fasting plasma glucose (95% CI) — mg/dl	98.15(98–98.29)	97.20(97.07–97.33)

*Abbreviation: HDL denotes high-density lipoprotein; LDL denotes low-density lipoprotein. To convert plasma glucose value to millimoles per liter, multiply by 0.0555. To convert low-density lipoprotein and high-density lipoprotein cholesterol to millimoles per liter, multiply by 0.0259. To convert triglycerides to millimoles per liter, multiply by 0.0113. Body mass index is calculated as weight in kilograms divided by the square of the height in meters.

**Table 2:** Standardized Prevalence of stroke and Vascular Risk Factors.

	No. of Participants	Prevalence of stroke % (95% CI)	Parental History of Stroke % (95% CI)	Systolic Blood Pressure mm Hg (95% CI)	LDL Cholesterol mg/dl (95% CI)	HDL cholesterol mg/dl (95% CI)	Triglyceride mg/dl (95% CI)
Total	207323	2.08(2.02–2.13)	6.31(6.2–6.41)	129.96(129.89–130.03)	111.73(111.59–111.87)	56.87(56.77–56.97)	11.48(11.34–11.63)
**Gender**							
Male	94913	2.38(2.29–2.47)	5.7(5.55–5.85)	130.17(130.07–130.28)	110.6(110.4–110.8)	55.47(55.32–55.63)	12.76(12.53–13)
Female	112410	1.82(1.75–1.90)	6.82(6.67–6.97)	129.78(129.67–129.88)	112.7(112.51–112.88)	58.07(57.93–58.21)	10.27(10.09–10.45)
**Age(years)**							
40–49	61383	0.64(0.58–0.7)	5.3(5.12–5.48)	124.97(124.86–125.08)	108.77(108.52–109.01)	56.53(56.34–56.72)	10.83(10.57–11.1)
50–59	58416	1.74(1.63–1.85)	7.67(7.45–7.89)	130.48(130.35–130.61)	114.29(114.03–114.55)	57.11(56.91–57.3)	12.88(12.59–13.18)
60–69	51695	3.87(3.7–4.04)	7.6(7.36–7.83)	135.15(134.99–135.31)	113.67(113.39–113.95)	56.45(56.24–56.65)	12.12(11.82–12.42)
70–79	27483	5.01(4.74–5.28)	5.1(4.83–5.36)	137.62(137.4–137.84)	112.4(112.01–112.79)	57(56.71–57.29)	11.03(10.64–11.42)
≥80	8346	4.62(4.16–5.08)	2.9(2.53–3.26)	135.65(135.26–136.04)	111.1(110.43–111.77)	57.35(56.84–57.86)	6.83(6.27–7.38)
**Residence**							
Urban	114999	1.9(1.83–1.98)	6.74(6.59–6.89)	128.94(128.85–129.03)	111.41(111.23–111.6)	56.25(56.12–56.37)	11.19(11–11.39)
Rural	92324	2.29(2.2–2.38)	5.8(5.64–5.95)	131.25(131.13–131.36)	112.13(111.92–112.33)	57.66(57.49–57.83)	11.84(11.62–12.06)
**Educational level**							
Low	76455	2.5(2.38–2.63)	6.17(5.94–6.4)	134.73(134.53–134.92)	111.17(110.84–111.51)	55.18(54.97–55.38)	11.36(11.04–11.67)
Medium	108140	2.22(2.1–2.33)	8.64(8.41–8.87)	129.41(129.27–129.55)	110.55(110.3–110.81)	55.17(54.99–55.35)	11.56(11.3–11.83)
high	22728	1.94(1.68–2.21)	9.89(9.33–10.45)	125.44(125.2–125.67)	111.43(110.91–111.95)	54.67(54.3–55.03)	12.33(11.72–12.94)

*Abbreviation: HDL denotes high-density lipoprotein; LDL denotes low-density lipoprotein. To convert low-density lipoprotein and high-density lipoprotein cholesterol to millimoles per liter, multiply by 0.0259. To convert triglycerides to millimoles per liter, multiply by 0.0113.

### Geographical Variations

**Figure 2:**
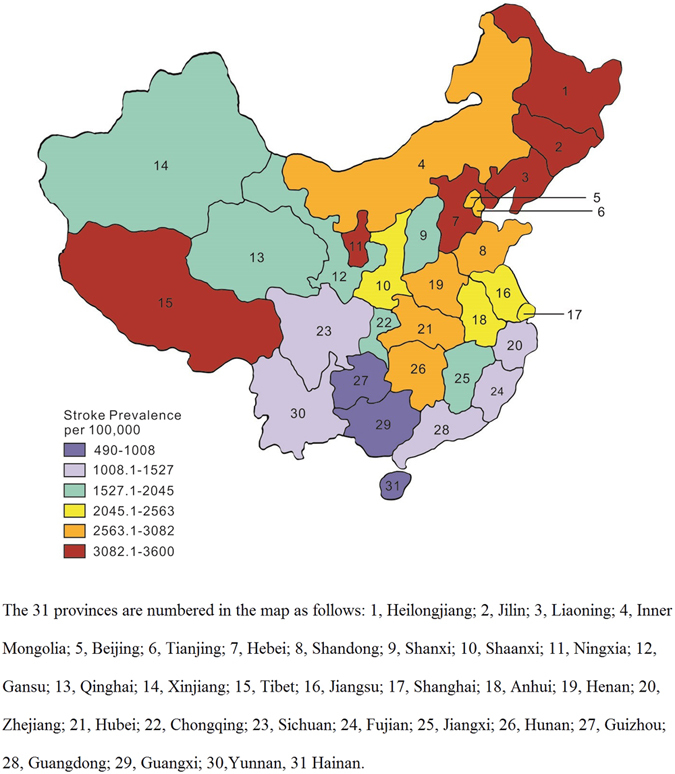
Map showing Age-standardized Prevalence of Stroke Across 31 Provinces in China. The colored map was drawn using Adobe Illustrator CS 6 (Adobe, California, USA).

The prevalence of stroke in the 31 provinces of Mainland China is illustrated in Fig. 2. The age-standardized prevalence of stroke was highest in Jilin province (3.6%, 3600 per 100 000) and lowest in Guangxi province (0.49%, 490 per 100 000). The age-standardized prevalence of stroke varied more than 7 fold among different geographic regions. The 31 provinces were geographically divided into northern and southern China using Huai River–Qin Mountains Line. The northern provinces include: Heilongjiang, Jilin, Liaoning, Inner Mongolia, Beijing, Tianjing, Hebei, Henan, Anhui, Shandong, Shanxi, Shaanxi, Gansu, Ningxia, Qinghai and Xinjiang. The southern provinces include: Jiangsu, Zhejiang, Shanghai, Chongqing, Sichuan, Fujian, Jiangxi, Hubei, Hunan, Tibet, Guizhou, Guangzhou, Guangxi, Hainan and Yunnan. There is a striking north to south gradient of stroke prevalence in China. The overall age-standardized prevalence of stroke was 2.51% in northern provinces, 2.92% in men, and 2.17% in women. The overall age-standardized prevalence of stroke was 1.51% in southern provinces, 1.68% in men, and 1.37% in women. The prevalence of stroke was significantly higher in northern provinces than in southern provinces in both men and women (P < 0.001). This trend was consistent in all age groups and in urban and rural populations.

### Stroke Risk Factor Burden

The prevalence of vascular risk factors showed a pattern similar to that of stroke, with greater burden of risk factors in the older age groups. Hypertension is the most common vascular risk factor among Chinese people aged ≥40 years. The overall age-standardized prevalence of hypertension was 35.24% in Chinese adults aged ≥40 years, 34.93% in men, and 35.42% in women. The overall prevalence of hypertension showed a similar inverse association with educational levels, with rates of 41.41%, 37.08%, and 32.41% in low-, middle-, and high-educational groups, respectively. The prevalence of dyslipidemia in the general population was 58.72% overall, 57.29% in men, 59.74% in women. The standardized prevalence of diabetes was estimated to be 9.55%, 9.62% in men, and 9.48% in women. Atrial fibrillation was observed in 1.57% of the general population aged 40 years and older. The mean systolic blood pressure and fasting plasma glucose all increased with increasing age in people < 80 years of age.

The lifestyle and metabolic risk factors are also prevalent in the general population. The overall age-standardized prevalence of smoking, alcohol consumption, overweight and obesity were 15.44%, 13.43%, 28.7% and 4.09%, respectively. In general, the prevalence of diabetes mellitus, smoking, alcohol consumption were significantly higher in men than in women (P < 0.001). However, the prevalence of hypertension, dyslipidemia, atrial fibrillation and obesity were significantly higher in women than in men (P < 0.001).

## Discussion

This large-scale, national community based study is the first to investigate the national burden of stroke and vascular risk factors in all 31 provinces in mainland China. Our study documented that 2.08% of Chinese adults 40 years of age or older have stroke, which equates to 11.8 million affected individuals. A door-to-door stroke survey^6^ of 63192 residents from six cities in 1983 showed that the age-adjusted prevalence of stroke was 719 per 100 000 and the prevalence ratio of completed stroke was higher in men than women (men: women = 1.5:l). A large scale population survey of 5 800,000 residents in 1986 indicated that the age-adjusted prevalence of stroke was 259.86 per 100,000 people^7^. Consistent with previous reports^17, 18^, we found that age-standardized prevalence of stroke was significantly higher in men than in women. A possible explanation is women smoke less and are likely to have fewer risk factors than men^18^. However, our study shows the higher prevalence of stroke in 2013 than that in 1983 and in 1986 in China. It turns out that stroke may have reached an alert level of epidemic proportions in the Chinese general population. In addition, hypertension, which is one of the most important risk factor for stroke, has affected approximately 19.8 million (35.24%) people of Chinese adults 40 years of age or older. Risk factors of stroke, such as hypertension, hyperglycemia, hyperlipidemia, have also been prevalent in the general population, with the potential for a major public epidemic of stroke if these risk factors are not under proper control.

In our study, a higher prevalence of stroke was observed in rural residents than in urban residents. In the past 20 years, China has experienced rapid urbanization, aging of population and drastic lifestyle changes. Urbanization is associated with lifestyle change that may lead to physical inactivity, great pressure of life and unhealthy diet, which may increase the risk of stroke^19^. However, urban residents may have higher socioeconomic level, better accessibility of health-care services and better knowledge and control of risk factors than rural residents^20, 21^. In addition, rural residents are adopting an unhealthy lifestyle due to industrialization in recent years. Together, these factors may lead to higher prevalence of stroke in rural residents.

We have noticed a substantial geographical variation in stroke prevalence in different regions in China. Overall, the prevalence of stroke was significantly higher in northern provinces than in southern provinces. The striking variation in stroke prevalence could be explained by the difference in the prevalence of hypertension between northern and southern provinces^22^. The 2002 national hypertension survey of 141892 Chinese adults ≥18 years has documented substantial geographic variation of the prevalence of hypertension in China^23^. A remarkable north-south gradient was observed and the prevalence of hypertension in the northern region of China almost doubled as compared with southern China provinces. The substantial variation in the geographic distribution of hypertension and stroke may be attributable to north-south gradient of dietary salt intake in China^24^. This pattern of hypertension is consistent with the geographic distribution of stroke prevalence reported in our study. Other factors, such as the cold temperature and dietary habits may also contribute to the geographic variation of stroke prevalence in China^25^.

Our study also showed an inverse association between education level and prevalence of stroke. We found that people with lower educational level may have higher prevalence of stroke in China. One explanation may be that high education is associated with good income and income is associated with resource availability. Highly educated people may have better access to preventative strategies and resources Educational level can be considered a good indicator of socioeconomic conditions^26^ and higher socioeconomic status is associated with lower incidence of stroke^27^. In addition, higher educational level has also been associated with better control of cardiovascular risk factors^28^. These findings may explain the inverse association between educational level and prevalence of stroke.

This study investigated the prevalence of stroke and associated vascular risk factors in the general population. The results of our study suggest that the burden of vascular risk factors is at alarmingly high level in China. Hypertension, dyslipidemia, diabetes, smoking and obesity are more prevalent in the Chinese population as compared with previous reports^29, 30^, which may explain the increasing prevalence of stroke in China. The increasing burden of vascular risk factors may be attributable to the rapid health transition in China as a result of aging population, rapid economic growth and urbanization^3^. The growing burden of stroke risk factors is particularly disturbing in China because the major cardiovascular events and case fatality in middle-income countries like China were higher than high-income countries^31^. Our findings underscore the need for implementing effective national primary prevention strategies that targets the modifiable risk factors for stroke.

In summary, the 2013 CSPP provides important data from 207323 participants across all 31 provinces in China. Our results show that the burden of stroke and its risk factors is huge and rapidly increasing in China. Stroke has become a major public challenge in China and there is an urgent need for development of national strategies for effective surveillance, prevention and management of stroke and vascular risk factors.

### Limitations

In this study, we focused on the burden of stroke and vascular risk factors. However, this was a cross-sectional study and only analyzed the prevalence of stroke, without considering the incidence, mortality, disability rate and recurrence rate of stroke. Therefore, the analysis of the burden of stroke was not thorough and comprehensive. Future studies are needed to further clarify the incidence, mortality and case-fatality of stroke in China.
